# Phosphorylation of Tau Protein as the Link between Oxidative Stress, Mitochondrial Dysfunction, and Connectivity Failure: Implications for Alzheimer's Disease

**DOI:** 10.1155/2013/940603

**Published:** 2013-07-10

**Authors:** Siddhartha Mondragón-Rodríguez, George Perry, Xiongwei Zhu, Paula I. Moreira, Mariana C. Acevedo-Aquino, Sylvain Williams

**Affiliations:** ^1^Douglas Hospital Research Center, Department of Psychiatry, McGill University, Montreal, QC, Canada H4H 1R3; ^2^UTSA Neurosciences Institute and Department of Biology, College of Sciences, University of Texas at San Antonio, San Antonio, TX 78249, USA; ^3^Department of Pathology, Case Western Reserve University, Cleveland, OH 44106, USA; ^4^Center for Neuroscience and Cell Biology, University of Coimbra, 3000-354 Coimbra, Portugal; ^5^Faculty of Medicine, Institute of Physiology, University of Coimbra, 3000-548 Coimbra, Portugal; ^6^Faculty of Medicine, Université de Montreal, QC, Canada H3C 3J7

## Abstract

Alzheimer's disease (AD) is defined by the concurrence of abnormal aggregates composed of phosphorylated tau protein and of abnormal cellular changes including neurite degeneration, loss of neurons, and loss of cognitive functions. While a number of mechanisms have been implicated in this complex disease, oxidative stress remains one of the earliest and strongest events related to disease progression. However, the mechanism that links oxidative stress and cognitive decline remains elusive. Here, we propose that phosphorylated tau protein could be playing the role of potential connector and, therefore, that a combined therapy involving antioxidants and check points for synaptic plasticity during early stages of the disease could become a viable therapeutic option for AD treatment.

## 1. Introduction

**Figure 1:**
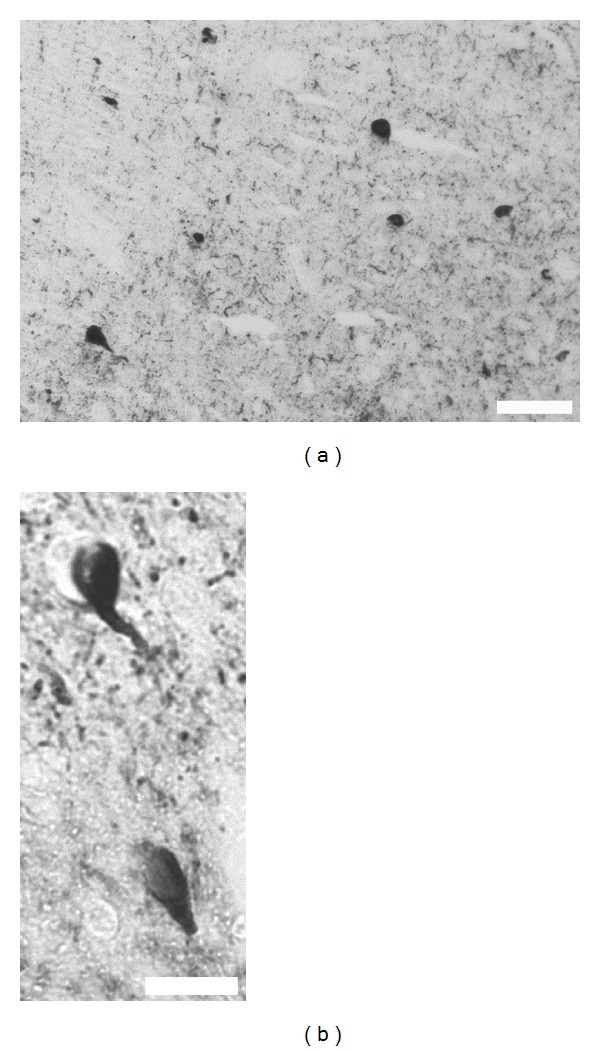
Neurofibrillary tangles are the classical hallmark of AD. Immunohistochemistry technique that evidences the most typical NFT that appears around affected areas (a, b). Here phosphorylated tau protein is the main element in the NFT aggregate scale bars 100 and 20 *μ*m, respectively.

Oxidative stress is the damage resulting from reactive oxygen species (ROS) that breach oxidant defences [1]. It has been found to play a crucial role during the development of many pathophysiological conditions including cancer, diabetes, cardiovascular disease, and neurodegenerative disorders [2–4]. Data has shown that oxidative stress is present in increased proportions during aging, similar to the increased susceptibility seen during Alzheimer's disease (AD) [5]. It has been strongly linked to neuronal dysfunction and ultimately to neuron death. It has also been suggested as a central mediator of toxicity [6], and the list will grow as bibliography is added to the discussion. In sum, evidence that links AD and oxidative stress is vast and continues to grow; however, a detailed mechanism leading from one event to the other remains elusive. In this regard, our group suggests that under degenerative conditions, the capacity of cells to maintain redox balance decreases resulting in mitochondrial dysfunction, metabolic dysfunction, deregulation of metal homeostasis, and alterations in the cell cycle [7–10]. Additional studies have shown that reactive oxygen species generated by mitochondria regulates p53 activity, which in turn regulates cell cycle progression and DNA repair and, in cases of irreparable DNA damage, executes programmed cell death [11]. This series of events certainly contributes to the classical fibril aggregations seen during AD, the neurofibrillary tangles (NFTs), mainly composed of phosphorylated tau protein (Figure 1). The role of aggregated tau protein during AD goes further: tau accumulation within the neuronal cytoplasm is associated with impaired axonal transport of mitochondria between the cell nucleus and synapse, which leads to severe energy dysfunction and imbalance in the generation of reactive oxygen species (ROS) and nitrogen species (RNS) [12, 13], all together leading to synaptic failure, another classical hallmark of AD and neurodegeneration [14]. Without discussion and according to current data, we can conclude that oxidative stress, protein deposition, and synaptic failure are crucially involved during neurodegeneration and specifically during AD; however, the order in which they appear during the progression and the identity of the mechanism that links them remains under extensive study. Addressing this point, evidence supports the hypothesis that mitochondrial and metallic abnormalities are direct precursors of oxidative stress during the early stages of AD [7, 15, 16]. In the same context we have proposed that deposition of tau protein could be a consequence of early posttranslational modifications like abnormal phosphorylation events [17]. In addition, we recently found that those abnormal phosphorylation events could be the link between synaptic failure and tau pathology [18], but more importantly, that those events could be occurring during early stages of AD (unpublished data). Therefore, taking this background into account, the purpose of this brief essay is not to discuss the effects of the disease, but rather to focus on chronology and mechanism of early events. With this in mind, the final objective is to identify the common mechanisms that could link all the events and therefore become an attractive therapeutic target for AD treatment. 

## 2. Mitochondria and Oxidative Stress: The Relationship

Since the brain is characterized by high energy consumption, mitochondria become crucial as an energy source. The role of mitochondria goes further; they are known for mediating anabolic/catabolic processes in several cell types, as well as for controlling a wide range of cellular processes, including cell proliferation and aging [19]. Unfortunately, under certain conditions, the production of free radicals [hydrogen peroxide (H_2_O_2_), hydroxyl (*·*OH), and superoxide (O_2_^−^·)], in which the brain is especially vulnerable, is an undesirable consequence [1, 20]. The reactive oxygen species, generated by mitochondria, have many targets such as lipids, protein, RNA, DNA, and mitochondrial DNA (mtDNA), which, due to the lack of histones, becomes a vulnerable target of oxidative stress [16]. Clearly, evidence supports the close dependence between mitochondria and brain activity, and because of this, it is therefore not surprising that oxidative stress as a product of mitochondria activity is related to neurodegeneration and, specifically, to AD. In this regard, during AD, significant reduction of intact mitochondria, as well as a reduction in microtubules, was found [21]. Oxidative stress markers, mtDNA deletion, and abnormalities in mitochondrial structure in the vascular walls of AD cases were also found to be increased [22, 23]. Changes in mitochondrial enzymes, mitochondrial structure, localization, and mobility are all involved in AD. In addition, markers for mitochondrial fission and fusion, which impair mitochondrial function, leading to energy hypometabolism and elevated reactive oxygen species production, are altered in models of AD [24]. It has been also reported that the neurons exhibiting increased oxidative damage in AD are coincident with striking and significant increase in cytochrome oxidases and mtDNA [25]. In sum, mitochondria contribute to AD, mainly through an oxidative stress-dependent mechanism with many targets in which tau is not an exception. Although the mechanism that links oxidative stress and tau has yet to be disclosed, recent data suggest that phosphorylation of tau protein could be the potential connector.

## 3. Phosphorylation of Tau Protein during AD

Formation of intracellular neurofibrillary tangles is one of the pathological hallmarks that characterize Alzheimer's disease (Figure 1). According to current AD hypotheses, (a) tau becomes abnormally phosphorylated, (b) dissociates from microtubules, and (c) aggregates into neurofibrillary tangles (NFTs) [26, 27]. Tau has more than 45 phosphorylation sites, most of which are located in the proline-rich region (P-region) (residues 172–251) and the C-terminal tail region (C-region) (residues 368–441) [28]. Tau phosphorylation at both of these regions affects its capacity to interact with microtubules [29]. Importantly, it is well documented that phosphorylation could contribute and enhance tau polymerization [30]. Beyond the vast and growing evidence that relates phosphorylation and aggregation during AD, we have found that phosphorylation of tau protein is probably the earliest event that occurs during tau abnormal processing in AD and other tau pathologies [17, 31, 32]. Additionally, we have found that phosphorylated tau plays a crucial role during synaptic plasticity, specifically during long-term depression (LTD) [18]. Interestingly, we reported that phosphorylation of tau protein is the key mechanism that regulates such a tau synaptic function [18, 33, 34]. So far, phosphorylation of tau protein seems to be an early phenomenon that relates to synaptic transmission. It remains to be determined, however, whether the phosphorylation of tau protein is an event that follows synaptic failure or if synaptic failure follows abnormal tau phosphorylation. The answer is certainly not straightforward, as it seems that both events could have a previous event. This is where oxidative stress could be a critical participant. Emerging evidence suggests that diffuse phosphorylated tau could be the result of endoplasmic reticulum stress, and vice versa, creating a pathological feedback loop [35]. We have also found that levels of tau phosphorylation are altered under oxidative stress conditions (unpublished data). Due to the multiple targets for oxidative stress, it is not surprising that it could affect tau phosphorylation levels. However, the fact that phosphorylation events in tau protein and synaptic transmission could be connected by oxidative stress becomes crucial. 

## 4. Tau Phosphorylation during Synaptic Transmission

Memory storage is probably one of the most intriguing questions currently being studied in the neuroscience community. Although the answer seems far from being fully addressed, growing evidence is helping to understand this complex and fascinating mechanism. At the cellular and molecular level, long-lasting synaptic plasticity is gaining acceptance as a model for memory storage. Synaptic strength can be long-lasting enhanced (long-term potentiation, LTP) or long-lasting depressed (long-term depression, LTD), and these changes can persist from seconds to hours and days [36, 37]. LTD is mediated by persistent presynaptic and postsynaptic changes. Importantly, such changes could suggest structural modifications that at present are not well understood. One of the best described mechanisms for LTD comprises N-methyl-D-aspartate (NMDA) receptor activation at many different synapses in the brain. This allows the entrance of Ca^2+^ to the cell and leads, through a Ser/Thr protein phosphatase cascade (GSK3*β*, PP1, Fyn, etc.), to removal of AMPA receptors from the postsynaptic membrane. The mechanism goes further, with caspase-3 being required for AMPA receptor endocytosis and LTD induction, in which cytochrome-C release from mitochondria is necessary for the activation of caspase-3 [38, 39]. At this point, the data establish the role for mitochondria during synaptic depression, but what could link this phenomenon to AD development? We recently showed that endogenous tau is found at postsynaptic sites where it interacts with the PSD95-NMDA receptor complex. That NMDA receptor activation leads to a selective phosphorylation of specific sites in tau, regulating the interaction of tau with Fyn and the PSD95-NMDA receptor complex, suggesting that abnormal NMDA receptor overexcitation could lead to abnormal tau phosphorylation, and therefore affecting synaptic transmission [18, 33, 34]. Overall, the data discussed here suggest that synaptic transmission is an indirect link between mitochondria and tau phosphorylation. 

## 5. From Single Neuron to Network Function: The Chronologic Point of View

**Figure 2:**
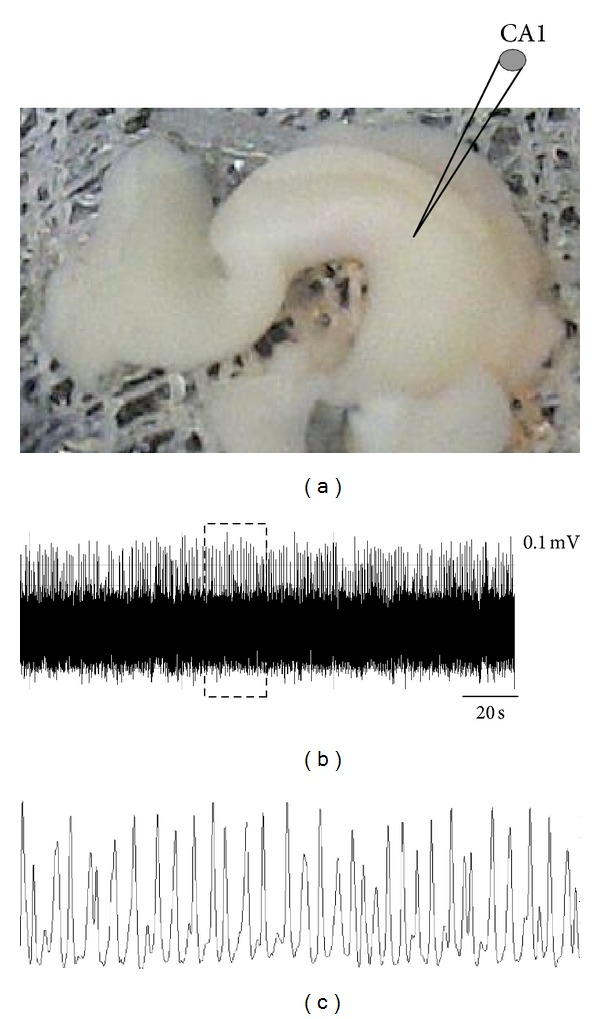
Dendritic recording of theta oscillations *in vitro*. Continuous recording (CA1 pyr layer) in the complete septohippocampal preparation (a). Voltage-dependent theta oscillation in pyramidal cell dendrites *in vitro* (b); see magnification in (c). The local field potential (i.e., the extra cellular potential measured in that brain area) shows robust and sustained oscillations at 2–4 Hz. This frequency is the theta band.

We have discussed data that tends to support the hypothesis of interconnection between oxidative stress and phosphorylation events in tau protein, events that mainly occurred during early stages of the neurodegeneration process; however, the early stage of synaptic failure has yet to be disclosed. In this regard, early memory changes, as the most related AD mark becomes key. Unsurprisingly, clinical AD symptoms are preceded by important network alterations. At this level, collective actions of neurons can be studied by recording theta oscillations in the brain [40]. Oscillatory activity in the theta range (3–12 Hz) is a dominant, synchronous signal in the hippocampus that has been shown to be present particularly during exploratory activity (Figure 2). Interestingly, it is now known that theta activity is an essential component of spatial memory and that its disruption leads to severe memory impairment [41]. Although the mechanism remains to be revealed, it is known that NMDA receptors, along with other receptors and transmitters, play a crucial role in theta oscillations [42]. Theta oscillations have been observed in numerous cortical structures: the subicular complex, entorhinal cortex, perhinal cortex, cingulated cortex, and the amygdale, among other structures present in the hippocampus [42]. Beyond the hippocampus as the main theta generator structure, the medial septum-diagonal band of Broca (MS-DBB) has also been proposed to act as a theta generator [43]. Recently, by using the complete septo-hippocampal preparation [44] from AD transgenic model (Figure 2), we found that synaptic connectivity between the MS-DBB and hippocampus is affected when compared to controls, but, more importantly, this event is present without evident cytopathology (unpublished data). Although the mechanism that links single molecular events and network connectivity remains under extensive study, this data, along with existing data, places synaptic transmission as an early phenomenon in which phosphorylated tau through NMDA receptors is certainly playing a crucial role with profound implications for network function involved in neurodegeneration.

## 6. Discussion and Perspectives

**Figure 3:**
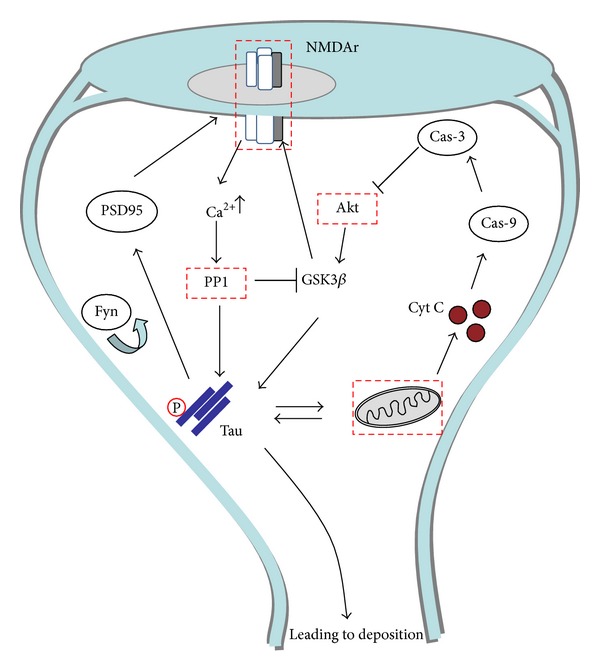
The relationship between synaptic transmission, tau, and mitochondria. The intricate and close relationship between mitochondria and synaptic check points makes them potential therapeutic targets (red squares). Phosphorylated tau protein acts as a central connector between mitochondria and synaptic formations. Calcium (Ca^2+^) enters via NMDA receptors and this leads to activation of protein phosphatase 1 (PP1), a key enzyme in synaptically-induced LTD. PP1 can dephosphorylate GSK3*β*, leading to the activation of tau and NMDA receptor. Tau contributes to NMDA activation through the PSD95-Fyn complex. Importantly, GSK3*β* is also activated by Akt through mitochondrial pathways, suggesting a fine regulatory mechanism. During neurodegeneration, oxidative stress affects levels of tau phosphorylation and GSK3*β*; this breaks the fine balance that controls memory formation, therefore leading to synaptic failure.

The main idea in this essay was not to discuss the role or relationship between oxidative stress and synaptic failure with AD, but rather to discuss the potential mechanistic relationship between them. In this regard, we propose that phosphorylated tau protein could be playing the role of a potential connector. Phosphorylation of tau protein seems to be the mechanism that regulates the physiological role of the protein at the synaptic terminal. Here, our data suggests that a fine balance of phosphorylation levels determines whether tau protein contributes to synaptic formation or neurodegeneration (Figure 3). But what could be causing such deregulation? Data lends support to the notion that oxidative stress is critically involved. Oxidative stress models, either *in vitro* or *in vivo*, are characterized by the presence of abnormally phosphorylated tau, in this direction, through inhibition of glutathione synthesis in M17 neuroblastoma cells, increased levels of tau phosphorylated were reported [45]. In null mice lacking superoxide dismutase whose phenotype is characterized by mitochondrial dysfunction and oxidative stress, abnormally phosphorylated tau was also found [46]. In sum, data tends to suggest that oxidative stress caused at synaptic terminals by mitochondria could be affecting downstream targets that have direct repercussion in tau phosphorylation levels, for example, GSK3*β*. Interestingly, altered tau can create a pathological feedback loop with mitochondria. This loop therefore creates a series of undesirable events for the neuron: (a) tau is no longer able to bind to PSD95, (b) tau does not recruit Fyn kinase that is crucial for NMDA receptor activation, and therefore synaptic formation is affected. Instead, tau is capable of affecting mitochondria that, in turn, will cause more damage to the neuron (Figure 3). At the end, the combination of all these events will result in spine retraction and synaptic failure. Therefore, therapeutic strategies that focus on early events, such as those described here, rather than on consequences such as protein deposition, will certainly offer a better possibility of success, as we previously discussed [31, 33]. Overall, our data, along with current data, suggests that tau abnormal phosphorylation could be a consequence of oxidative stress, with this relationship having critical repercussions for synaptic transmission. Therefore, a combined therapy that considers antioxidants and check points for synaptic plasticity during early stages of the disease could offer new hope (Figure 3).

## Abbreviations

ROS: Reactive oxygen species
AD: Alzheimer's disease
NFTs: Neurofibrillary tangles
LTD: Long-term depression
NMDA: N-Methyl-D-aspartate receptor.
